# ColonyInsight automated workflow reveals complex dominance in mixed yeast colonies

**DOI:** 10.1016/j.isci.2026.116639

**Published:** 2026-07-22

**Authors:** Tünde Gaizer, Bence T. Gaizer, Bíborka Pillér, Valentina Madár, János Juhász, Attila Csikász-Nagy, Csaba I. Pongor

**Affiliations:** 1 Pázmány Péter Catholic University, Faculty of Information Technology and Bionics, Budapest, Hungary; 2 Semmelweis University, Institute of Medical Microbiology, Budapest, Hungary; 3 Cytocast Hungary Kft, Budapest, Hungary

**Keywords:** microbial interactions, yeast, *S. cerevisiae*, colony growth, co-culture, high-throughput, fluorescent imaging

## Abstract

We present ColonyInsight, a scalable workflow for quantifying microbial interactions in solid phase cultures. Using four *Saccharomyces cerevisiae* strains tagged with fluorescent markers, we combined time lapse imaging with growth curve analysis to characterize competitive dynamics under different environmental conditions. ColonyInsight integrates a cost effective imaging platform with a dynamic time warping based metric to compare colony growth and infer dominance relationships. Validation by fluorescence microscopy showed that growth based measures reliably estimate competitive outcomes. Applying this approach revealed environment dependent dominance hierarchies across strains in rich and minimal media. ColonyInsight enables systematic, high content analysis of microbial interactions and provides a flexible framework for studying dominance and community behavior across diverse microbial systems.

## Highlights

• Cost-effective imaging station automates acquisition of high content colony data
• Growth curve similarity estimates dominance in mixed yeast colonies
• Dominance hierarchies among yeast strains shift between rich and minimal media
• Mixed yeast colonies show diverse spatial organization and complex niche partitioning

## Graphical abstract

**Figure:**
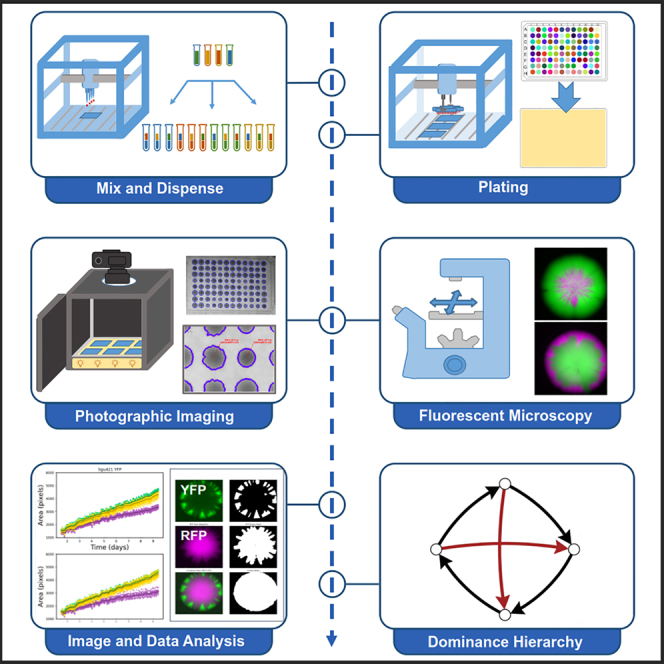

## Teaser

Biological sciences; Microbiofilms; Microbiology

## Introduction

Microbial life is shaped by a complex interplay of environmental factors and community level interspecific interactions as they inhabit diverse ecological niches. These interactions are fundamental drivers of community structure and function defining their ecological role from natural habitats like the soil^1^ or our gut^2^ to more industrial settings in the food and wine industry.^3^ Elucidating how these complex interactions shape community assembly and govern metabolic and signaling networks is a central goal in microbiology.^4^ While traditional microbiological methods have provided invaluable insights into the behavior of microbial communities, the advent of automation in the laboratory has given us ever expanding possibilities to advance our capacity to better understand these interactions. Most current workflows for high-throughput experimentation have been developed to study well mixed liquid cultures.^5^^,^^6^^,^^7^ Although liquid phase studies are invaluable and are biologically relevant, they preclude the study of spatially structured communities typically found in nature on solid media. In surface-associated habitats, microbial populations undergo range expansion where they compete for space, are frequently embedded within an extracellular matrix, and are exposed to steep physico-chemical gradients of oxygen, nutrients, and metabolic products. Furthermore, these conditions facilitate contact-dependent signaling and antagonism, all of which are critical factors shaping community structure and function that are fundamentally different in liquid media. Directed studies of interactions between specific strains or organisms were performed on solid media, but these are not scaled for large scale studies of interactions in mixed colonies.^8^ Most existing high-throughput methods developed to study microbial interactions on solid phase focus on intra-colony dynamics^9^^,^^10^^,^^11^^,^^12^ or use microbial lawns to screen interactions, these cannot fully capture how mixed communities behave.

In this work, we developed a highly automated workflow to acquire high-content information on interactions between unlabeled and fluorescently labeled microbes in solid-phase co-cultures. The workflow can be easily scaled for high-throughput experimentation. This platform integrates automated sample preparation, a cost-effective imaging station and image processing workflow and an automated microscopy protocol. Time-lapse imaging is used to acquire high temporal resolution growth curves to compare the spatiotemporal dynamics and fitness of co-cultures relative to mono-cultures, while automated microscopy is used to assess the distribution of the different components within the colony biofilms. Specifically, in this study we have investigated the formation of two strain *Saccharomyces cerevisiae* co-colonies and found that there is a surprising variety in the dynamics of colony formation compared to typical microbial co-cultures.

## Results

**Figure 1:**
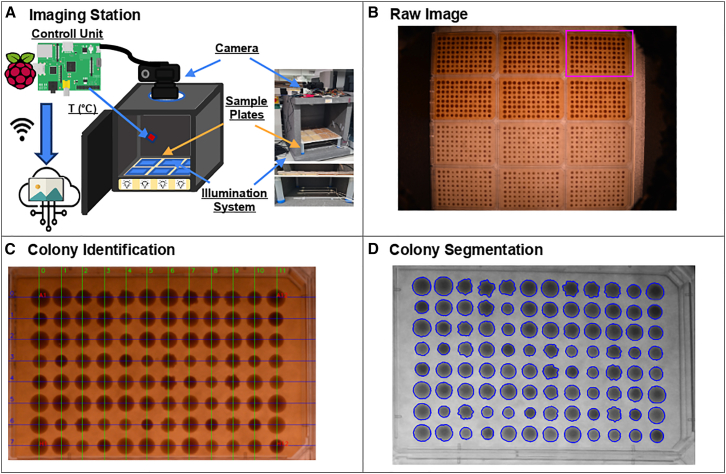
Overview of the ColonyInsight imaging and image analysis workflow (A) Schematic overview of the imaging station. (B) An example of a raw image taken by the imaging station is presented, showing the 12 Omnitray plates after 9 days. The plate highlighted with the magenta frame is shown on C. (C) Colony positions are identified in rows and columns on each plate separately. (D) Colonies at each position are segmented.

To facilitate high-throughput analysis of microbial interactions on solid-phase, we established an end-to-end modular workflow that we named as ColonyInsight. The pipeline integrates automated sample preparation with multi-scale imaging using a custom imaging station and an automated microscopy protocol. ColonyInsight begins with an automated sample preparation workflow to mix the strains pairwise and dispense samples onto multi-well arrays. Next, the liquid handling system utilizes a custom pinning array to inoculate high-density co-cultures onto solid media. These arrays are monitored using our cost-effective, custom imaging station (Figure 1 A) to capture growth kinetics via time-lapse photography. The goal is to extract a dominance hierarchy of interacting microbial strains (in our case baker’s yeast). Here, between two interacting partners, the dominant strain is taken as the strain that more strongly determines the growth characteristics of the co-cultured colony starting from a 1:1 mixture. From these results, we can extract an interaction network and a hierarchical ordering of different strains.

### Sample preparation

The strain interaction analysis workflow was tested on 4 strains that were selected to include both laboratory strains like Y55,^13^ BY4743,^14^ and wild strains like YPS606,^13^ Sgu421^15^ strains. We tagged each with constitutive YFP and RFP fluorescent markers. Co-cultures were created by mixing every YFP variant with every RFP variant in a 1:1 ratio. We plated mixed cultures in quadruplicates alongside pure monocultures, filling a standard 96 well array. In our experiments, we have plated the array on 12 plates using two different media (YPD and SD).

While one can prepare these samples by hand, manual processing is tedious, prone to error, and cannot be scaled for larger scale studies. To enable high-throughput analysis, we established an automated workflow using a Hamilton Microlab Starlet liquid handling system. The process splits into three distinct phases: mixing, dispensing, and pinning. We wrote the protocols in the Venus 4 environment with a simple UI to make the system accessible to everyday users. Users can easily customize the protocols using custom excel files to define mix ratios and plate layouts (see zenodo repository and supplementary materials).

For the final transfer step, the robot uses a custom pinning tool with 96 steel pins to inoculate onto solid media. Besides making the process easier, automated pinning is also essential for precise positioning. The protocol immerses the pins in the liquid medium twice to prevent bubble formation. Users can process three plates in a single cycle, and the system allows for washing the pins with isopropanol between sample changes. In the washing steps, pins are immersed in isopropanol for 3 min in 3 consecutive steps. All of these steps can be fully customized through the UI of the protocol. For further details, see Figure S1.

### Imaging setup

Previous imaging methods used photographic setups to study solid colonies and their growth commonly on single plates.^16^^,^^17^^,^^18^^,^^19^^,^^20^^,^^21^^,^^22^^,^^23^^,^^24^^,^^25^^,^^26^^,^^27^ These camera-based solutions are less adaptable and scalable for high throughput studies of colony growth. Another widely employed solution is the use of flatbed scanners to image plates.^28^^,^^29^^,^^30^^,^^31^^,^^32^^,^^33^^,^^34^ While this can be scaled, these systems are less user-friendly solutions when scaling and need a separate module to control temperature.

To acquire long-term, high-frequency growth data, we constructed a custom imaging station (Figures 1A and S2) which is cost effective and more easily scalable than previous solutions. Our method relies on an off-the-shelf camera (in our case a DSLR) to image a larger array of plates simultaneously. A difficulty in carrying out reproducible long term imaging experiments of culture plates is the control of temperature. The temperature fluctuations caused by daily fluctuations in temperature and the temperature gradient caused by the illumination source causes condensation on plate lids. To carry out the experiments we have built an enclosure from 50 mm thick expanded polystyrene (Styrofoam). The enclosure minimizes environmental instability, such as air currents and thermal fluctuations, and ensures optical isolation as well.

For image acquisition, we utilized a Nikon D3200 DSLR camera fitted with a standard 55–77 mm zoom lens. This optical configuration provides the necessary field of view and resolution to image 12 plates simultaneously (Figure S3). To achieve uniform background lighting without specular reflections, we designed a *trans*-illumination system using four LED strips positioned at the base of the enclosure. Samples are elevated on a 10 cm stand topped with an opaque glass plate and a layer of filter paper, which function as diffusers to maximize homogeneity. The LEDs are triggered via a transistor circuit, ensuring low power output to further mitigate heat generation.

Time-lapse images are acquired using a Raspberry Pi 5 single-board computer that runs Raspberry Pi OS (Debian 12, Bookworm). We developed a custom control script in Python 3 that utilizes the gphoto2 interface (available at: http://www.gphoto.org/ to synchronize image capture with illumination (see zenodo repository and supplementary materials). Additionally, a DS18B20 digital sensor is integrated to continuously monitor the internal temperature throughout the assay. Temperature was within +-1 °C following daily cycles of room temperature (Figure S12); no major warming from light source was measured, and no vapor condensation on the lids was observed in any of our experiments.

### Processing of time-lapse images

The image processing pipeline must solve two questions. In the first step, the position of all the plates on the field of view must be identified and located. In the second, the colonies on each plate must be identified to extract colony area and circularity. The image processing pipelines have been implemented in Python.

The pipeline to identify the plates uses the first images (without visible colonies) to identify individual plates (see Figure S3). First, it transforms the image to grayscale and uses light equalization to remove artifacts from uneven lighting. Next, it applies a threshold to find segments of the plate borders, then employs a distance transform followed by another thresholding to select central area of each plate (seeds). From the seeds identified in the previous step, the border of the plates is identified using a ray-tracing algorithm. Our current implementation is optimized to identify only rectangular plates that are positioned in a grid pattern.

After all the plates have been located, the last image of the experiment is analyzed to locate colonies on the culture plates (see Figures 1B–1D). The grid pattern constituted by the colonies is located using the sum of intensities in all rows and columns using the Fourier transform. A region of interest (ROI) is created at each grid location; a smoothing operator is applied, and the largest peak is located. If the identified peak clears a predefined threshold, the ROI is deemed to have a colony within its bounds. Finally, we use the difference of Gaussians (DOG) method to accurately locate colony borders. This analysis is run for each image in the timeline to determine intensity and size parameters. Tabular data are exported for further analysis and saved.

### Processing growth data

**Figure 2:**
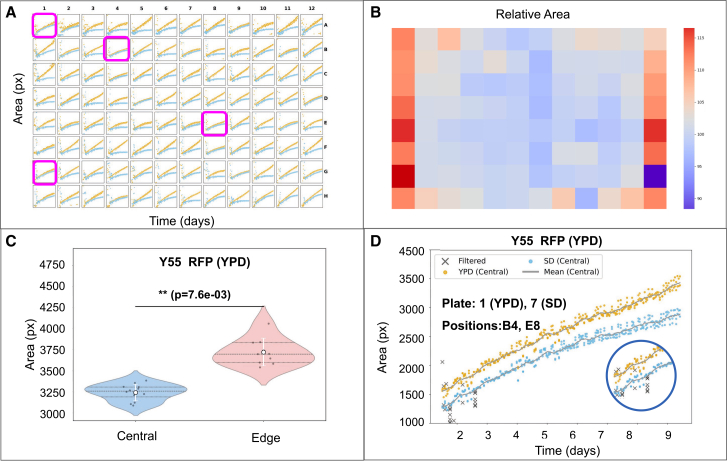
Raw growth data and data preprocessing (A) Raw growth curves (area in pixels/px) extracted from a YPD (Yellow) and SD (Blue). Each plot corresponds to a single colony from one SD and one YPD plate. All instances of Y55 RFP are highlighted with magenta frame, these positions are analyzed on C and D. (B) Heatmap representing the relative area of colonies compared to the central colonies’ mean area. For each strain/strain mixture: the mean of the area was calculated for colonies in the central area and used to normalize each value. Values represent the mean across all replicate plates (*n* = 6 per environment) rendered on a logarithmic color scale. Values above 1 (warm colors) indicate larger-than-central growth; values below 1 (cool colors) indicate smaller-than-central growth. (C) Violin plot comparing colony area (pixels) at day 9 between central (blue, *n* = 12 colonies) and edge (red, *n* = 9 colonies) positions for the Y55 RFP strain, pooled across all replicate plates (*n* = 6, YPD). Central colonies comprise the mid and inner-ring positions; edge colonies comprise the outermost row and column positions. Individual data points represent single colonies. Groups were compared using Welch’s *t* test; ∗*p* < 0.05, ∗∗*p* < 0.01, ∗∗∗*p* < 0.001. Individual data-points represent the area of a single colony in pixels (px). (D) Pooled growth curve data for the Y55 RFP strain from colonies in the central region from one plate. Excluded points are shown as gray crosses, and mean curves are plotted with gray solid lines (see excerpt).

The imaging systems and image analysis pipeline generate raw tabular data that contains the area and circularity metric for all colonies on each plate (see Figure 2A) spanning the 12 plates that have been imaged.

Preprocessing of the growth curves includes cleaning from outliers and noise (Figure 2D). The first 1.5 days of the experiments are cast away, as in the early time-points colonies are barely visible, which causes the image processing algorithm to mistakenly detect noise. Next, we apply a spatial filter and only keep data from the central area due to edge effect (see Methods section for details). We then filter colonies with a circularity lower than 0.85 as measured using OpenCV^35^ as we expect nearly circular colonies based on this metric. The remaining data then undergoes a dual-pass percentage filter, first flagging local time-series artifacts deviating >15% from their 10-point rolling average, and subsequently removing peer-group dissenters straying >10% from the group mean. During the analysis, curves are also smoothed with a 10-point smoothing window (Figure 2D).

The edge effect is a known confounding factor in plate-based assays. Peripheral colonies grow at different rates as central ones as they have fewer neighbors to compete with for resources.^36^^,^^37^^,^^38^^,^^39^ We have examined the extent to which this is present to our dataset. Spatial heat-maps were generated by applying a normalization scheme to the samples (Figure 2B). In the normalization scheme, each sample colony area value was divided by a reference mean calculated from its corresponding central colonies. Following this correction step, the normalized data for all replicate plates was averaged. The resulting matrix, representing the average normalized value for each spatial position, was then rendered using a logarithmic color scale to visually map positional effects across the plate.

In order to quantify the edge effect, we have compared the distribution of colony areas in the central area and edge of each plate at the end of the experiment (day 9). The distributions of these two populations were visualized using violin plots (kernel density estimation) overlaid with individual colony data points (see Figure 2C). Finally, an independent two-sample *t* test was performed to quantify the statistical significance of any observed differences between the means of the “Central” and “Edge” groups. In most cases there was a significant difference between the two populations (Figure 2C and Supplementary materials). Based on this analysis we have decided that further analysis would be necessary to reliably establish our understanding of edge-effect on mixed colony growth. To maintain robustness of the current work, we chose to exclude edge colonies from the quantitative analysis.

### Analysis of growth dynamics

**Figure 3:**
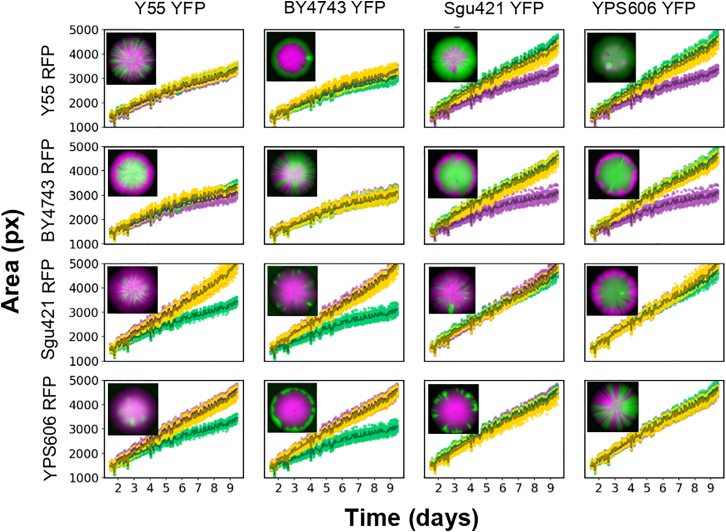
Overview of growth-curve data and microscopy images on YPD A comprehensive overview of competitive interactions between the four differently labeled yeast strains (Y55, BY4743, Sgu421, and YPS606), each labeled with either yellow fluorescent protein (YFP) or red fluorescent protein (RFP), grown on YPD. Each subplot corresponds to a specific pairing and within each subplot, scatter points show individual colony growth curves pooled across all replicate plates (*n* = 6 plates per condition; approximately 2–3 central colonies per plate per sample). The solid line represents the mean growth curve computed across all central colonies from all plates combined, smoothed with a 10-point centered rolling average. Each subplot also shows a selected two channel microscopy image after 9 days of growth where magenta corresponds to RFP and the green channel corresponds to the YFP signal.

To test and validate the method on mixed colonies of strains followed during colony growth, we analyze the growth dynamics of all pairwise co-cultures between four yeast strains (Y55, BY4743, Sgu421, and YPS606), each labeled with either RFP or YFP. A pair-plot is created for each environment where the columns represent strains with a YFP tag and rows represent RFP tagged strains and each grid element in the plot shows the growth curve of the pure YFP variant in green, that of the RFP variant in magenta and finally the growth curve belonging to the mixed colony in yellow (Figure 3 for YPD; Figure S4 for SD). The environment has a strong impact on the growth characteristics of the strains; the difference is especially strong for the Sgu421 and YPS606 strains. While on rich YPD their growth rate is easier observable, on SD their growth curves are flatter and reach a plateau much earlier (Figure S4).

On both YPD and SD media, the growth curve of a mixed colony typically followed that of the faster-growing mono-culture, indicating that the fitter strain dominates the overall growth dynamics. One example where the trend is the opposite on the SD environment where the otherwise slower BY4743 RFP seems to dictate the growth of the mixed colony for Sgu421 YFP (see Figure S4).

### Determining dominance hierarchies using growth dynamics

After establishing the automated pipeline for acquiring high resolution growth curves, the subsequent challenge was to translate the phenotypic properties of single and mixed cultures into biological interactions and to construct a dominance hierarchy of the strains. Comparing the growth curves of co-cultures we have found that the macroscopic growth trajectory of a mixed colony frequently follows the kinetics of the dominant, fitter strain. To systematically unravel competitive hierarchies without relying exclusively on fluorescent labeling, we chose to analyze growth curve similarity. This section details how quantifying the kinetic similarity between co-cultures and their respective mono-culture controls allows inference of strain dominance and the construction of interaction networks directly from solid-phase growth patterns. To get a quantitative and objective reference of which strain is dominant among the two strains that make up co-cultured strains, we continued to analyze the fluorescent microscopy images. Since we have labeled the strains with fluorescent markers, we can estimate the area within each colony where the two strains dominate. This can then be used to validate how much curve similarity can be used to predict dominance within the colony.

**Figure 4:**
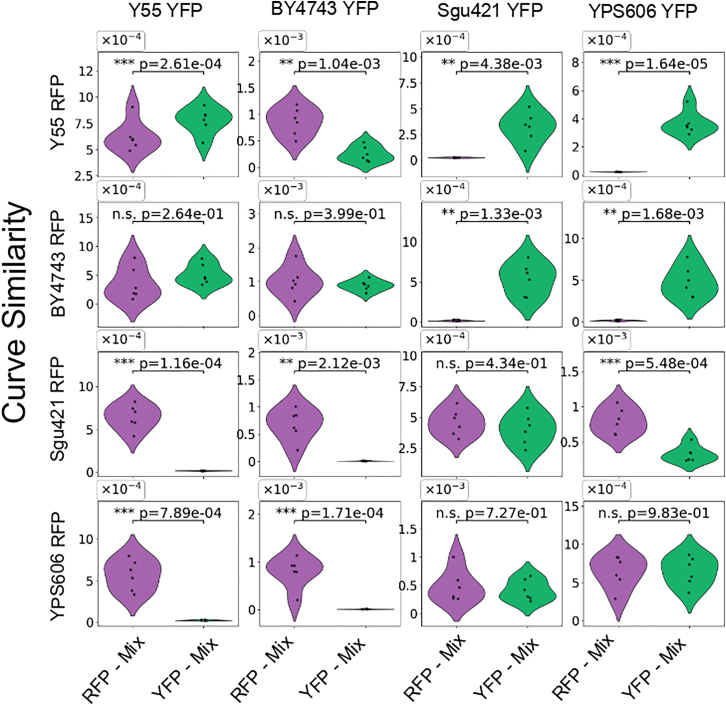
Overview of dynamic time warping (DTW) curve similarity metric (s) on YPD A comprehensive grid-plot visualizing DTW-based similarity metric. Each subplot corresponds to a specific pairing, with the row indicating the RFP-labeled strain and the column indicating the YFP-labeled strain. On each subplot the mixed colony’s growth curve is compared to the growth curve of both of its constituent strains. Each data point represents the DTW-based similarity score calculated between the per-plate mean growth curve of the co-culture and that of the respective monoculture (*n* = 6 replicate plates per condition). Per-plate mean curves were derived by averaging colony area across central colonies at each time point, followed by 10-point centered rolling average smoothing. Violin shapes show the distribution of per-plate similarity scores. Groups were compared using Welch’s *t* test; ns = not significant, ∗*p* < 0.05, ∗∗*p* < 0.01, ∗∗∗*p* < 0.001.

To quantify curve similarity of co-cultures with respect to the constituent mono-cultures we chose the dynamic time warping (DTW) algorithm.^40^ This method is often used to quantify the similarity of time series; it is resilient to phase shifts and magnitude-based differences that is inherent in biological systems and thus has also been applied to analyze the temporal dynamics of microbial systems.^30^^,^^41^^,^^42^^,^^43^ DTW essentially calculates the optimal alignment cost *d* between the smoothed single strain and mixed-culture curves (Figure 4 for YPD; Figure S5 in SD). This distance-based metric is transformed into similarity using the following transformation:

(Equation 1) $$s=\frac{1}{1+d}$$

where s is the similarity and d is the DTW distance metric. The DTW based similarity of the mixed curve is compared using an independent two-sample *t* test (Welch’s *t* test). On the diagonal we have combinations of the same strain with different labeling, and the DTW similarity distributions are typically statistically indistinguishable. The mixed colonies, on the other hand show higher variability. In some cases, growth dynamics strongly differed, and the mixed colony growth curve was indistinguishable from one component.

### Validation of dominance hierarchies through fluorescent imaging

**Figure 5:**
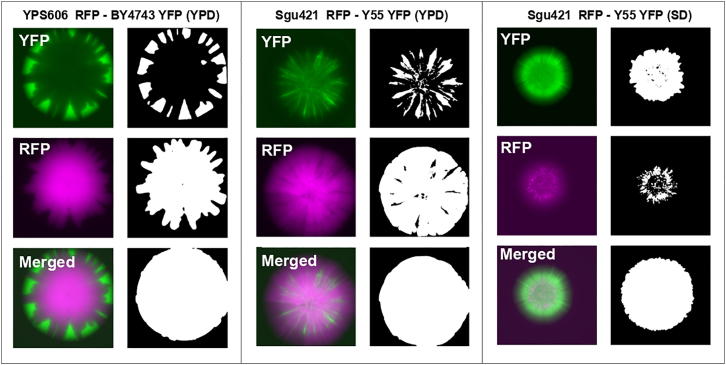
Segmentation of microscopy images Representative images showing raw microscopy images (Green:YFP, Magenta:RFP) to the left and segmented area to the right. The first row shows the green channel, the second row shows the magenta channel, and the third row shows the combined images and segmentation masks.

To validate the growth curve similarity-based prediction of the dominance of a given strain above another one, we have also taken microscopy images of the mixed colonies shown on Figure 3 (YPD) and Figure S4 (SD) inserts. Quantitative analysis of multichannel fluorescent microscopy images is a complex task due to both physical, chemical, and biological reasons. Additionally, the size of the colonies makes direct quantitative comparison difficult as even with a 2× objective the depth of field is not enough to encompass the whole thickness of the colony. These make it difficult to directly compare fluorescent intensities, thus we have devised an auto segmentation scheme to get a quantitative measure of the area occupied by each strain (see Figure 5 and Supplementary materials).

The workflow begins by defining a primary ROI for the entire colony. This global colony mask is generated: first low intensity background is removed by a percentile-based threshold to each of the two raw fluorescence channels (YFP and RFP), the cleaned grey-scale channels are then combined, binarized by thresholding and undergoes a morphological transformation to remove speckled noise. Once the global colony mask is established, the individual channels are also segmented separately. First, the uneven illumination is corrected and scaled by dividing the channel by a background map generated from a large-sigma Gaussian blur (sigma = 100). Finally, each strain’s territory is segmented using the moments auto-thresholding method as implemented in the SimpleITK package.^40^ Fluorescent imaging and segmentation were carried out for one plate in each condition.

Grid plots that summarize the relative area of the two strains can be seen in Figure S6 (for YPD) and Figure S7 (for SD), showing approximately 5% deviation. These plots quantify the dominance and compare the growth curve similarity based (Figure 4) and fluorescence-based data through a newly introduced dominance index (DI). This metric was defined for both the curve similarity metric and the relative area from fluorescent microscopy images as:

(Equation 2) $$DI_{similarity}=\log_{2}\frac{sGFP}{sRFP}$$

(Equation 3) $${DI}_{Fluorescent}=\log_{2}\frac{{Area}_{rel}YFP}{{Area}_{rel}RFP}\text{,}$$

where *s* is the DTW similarity and *Area_rel_* is the relative area within the colonies. A positive index signifies that the co-culture is dominated by the YFP strain, while a negative index indicates greater similarity to the RFP strain.

**Figure 6:**
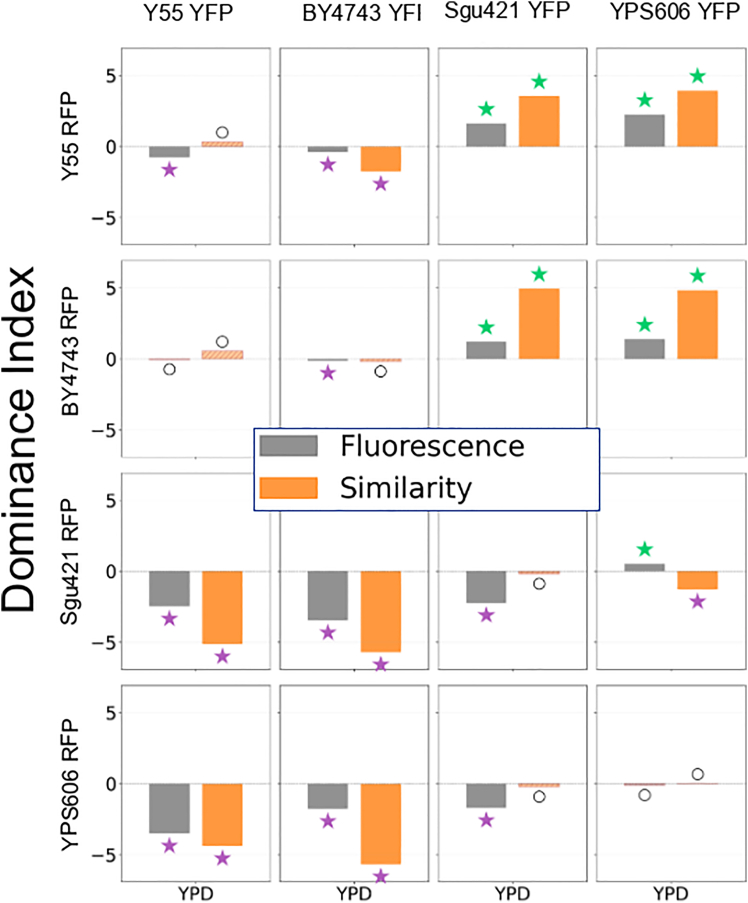
Grid plot for dominance indices on YPD Each subplot within this grid directly compares the dominance indices (fluorescence and similarity) for a single strain pair. The dominance index (DI) is calculated as the log2 ratio of either the growth curve similarity scores or the relative fluorescent areas of the competing YFP and RFP strains within a co-culture as defined in Equations 2 and 3. This visualization classifies each result based on the sign of the dominance index and interactions are deemed as neutral if there is no significant difference with *p* value>0.05 (Welch’s *t* test). A significant or above-threshold dominant strain is designated by a star symbol (★), while a neutral interaction is marked with an open circle (○). Negative values correspond to dominance by the RFP, positive values by the YFP expressing strain, represented by the colors of the significance stars.

To facilitate a detailed, pairwise comparison of competition outcomes based on curve similarity and relative area based on fluorescence, on Figure 6 we present a 4×4 grid of bar plots that visualize the DI for all 16 strain pairings. Each subplot within this grid directly compares the dominance indices for a single strain pair across all tested environments (Figure 6 for YPD; Figure S8 for SD). This plot classifies each result as dominance, based on the sign of the DI and interactions are deemed neutral if there is no significant difference between curve similarity or relative area, with *p* value>0.05 based on a Welch’s *t* test. The figures show that in most cases there is a good agreement between the similarity and fluorescence-based method. Often the disagreement is for cases where dominance indices are rather small.

To further evaluate how well the developed growth curve measurement data can properly capture how the competing fluorescently marked strains interact, we studied the correlation between the fluorescence-based and growth-curve-based dominance indices (shown on Figure S9). Results show that there is a strong correlation between the fluorescent and growth curve similarity dominance indices, with r > 0.85 for both environments. Overall, the two methods predict the same trend in 12 out of 16 pairings on YPD and 14 out of 16 cases on SD. This highlights that the introduced growth rate measurement and data analysis method can capture if one of two strains out-compete the other and thus give an estimate to the dominance hierarchy.

### Overview of dominance hierarchies

**Figure 7:**
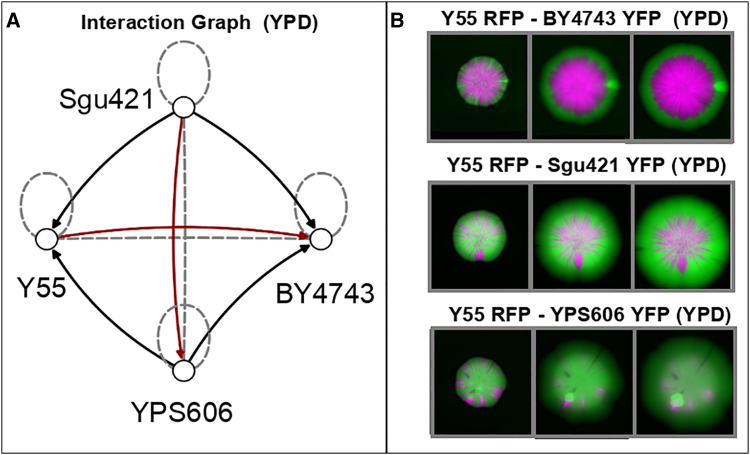
Summary of dominance hierarchies and spatial patterns on YPD (A) Interaction graph. Nodes represent interacting partners. The edges connecting the nodes visually encode the outcome of the competition. Directed arrows are drawn from the dominant strain to the less dominant partner. Neutral interactions are rendered as a gray dashed line, for cases where dominance indexes are indistinguishable (using *p* > 0.05, Welch’s *t* test). Black arrows indicate a consensus outcome where both reciprocal labeling combinations agree on the direction of dominance. Colored arrows (red or green) indicate cases where the reciprocal labeling combinations differ; in these instances, the edge color reflects the specific color of the dominant strain in that combination. (B) Representative microscopy images of mixed colonies. Images were taken at day 4, day 7, and day 9.

To visualize the competitive profile of a strain, we generated interaction graphs for each individual strain based on curve similarity (Figure 7 and Figure S10). The edges connecting the nodes visually encode the outcome of the competition. A directed arrow is drawn from the dominant strain to the less dominant partner-in cases where one strain dominates in the interactions. Neutral interactions are rendered as a simple gray dashed line. Loops represent interaction between the YFP and RFP variants of the same strain. If the two combinations differ, both edges are visualized and if the interaction is non-neutral the color reflects the color of the dominant strain.

## Discussion

Our main highlight from the interaction networks is that dominance is environment-dependent and is not a fixed trait. In the nutrient-rich conditions of YPD, we observed that Sgu421 and YPS606 acted as aggressors, defeating all challengers. However, their advantage decreased when nutrients were restricted in the minimal SD environment as shown on Figure S10. On SD, Sgu421 lost its footing, allowing YPS606, and Y55 to the top of the hierarchy. This reversal highlights a compelling ecological trade-off: the high-velocity growth strategies that secure victory in high nutrient environments. The laboratory strain, BY4743 consistently remained at the bottom of the dominance ladder.

### Internal structures of mixed colonies

Fluorescent images revealed that these interactions produce a surprising variety of complex spatial patterns (Figure 3; Figure S4). We can classify the observed outcomes in three groups. In the first, we see colonies with sectored, petal-like domains (see Y55 RFP vs. Y55 YFP on YPD in Figure 3). Second, we find core-ring structures where one strain seems almost to encapsulate the other (e.g., Figure 3: BY4743 vs. YPS606 on YPD or Figure S4: Sgu421 vs. BY4743 on SD). Pairs like BY-Y55 on and Sgu421-YPS606 on YPD or YPS606-Y55 on SD have a comparable growth rate yet still seem to segregate spatially, which indicates that the strains in fact do interact in a significant way and find distinct ecological niches. Pairs of YPS606-BY, Sgu421- BY4743, and YPS606- BY4743 on YPD, form ring-like patterns where the slower strains form an external ring structure. Remarkably, this does not incur overall fitness cost; the growth rate of the mixed colony was indistinguishable from that of the faster YPS606 mono-culture. This suggests that the slower strain thrives in the unique environment of the colony’s edge. The third distinct class is when one strain completely overgrows the other showing strong dominant behavior (e.g., Y55 vs. YPS606 on YPD or Sgu421 vs. Y55 on SD). These results demonstrate that even among sub species level competitive interactions lead to complex community structures.

Studies with respect to the spatial distribution of mixed microbial colonies on solid media^44^^,^^45^^,^^46^^,^^47^^,^^48^^,^^49^^,^^50^^,^^51^^,^^52^^,^^53^^,^^54^^,^^55^^,^^56^^,^^57^^,^^58^^,^^59^^,^^60^^,^^61^ show that the formation of colony biofilm patterns is driven by complex environmental, chemical, and genetic patterns. High throughput methodologies are crucial to understanding these effects. In this study we have developed at the high content ColonyInsight workflow that combines automated sample preparation, a cost-effective DIY imaging station and data analysis to study microbial interactions to systematically characterize microbial interactions in co-cultures on solid media. The ColonyInsight workflow is easily scalable, can be used to quantify dominance characteristics of a strain above another one, and validated through an automated microscopy method. ColonyInsight is also ideal to screen colony growth at various solid media sources for effects on microbial libraries and microbial interactions, as we have also observed major differences in interactions in YPD and SD.

We have applied ColonyInsight to gain deeper insights into interactions of four *S. cerevisiae* yeast strains on two different media: the rich medium YPD and the minimal medium SD. We have shown that we are able to record high resolution growth-curves of 12 plates with 96 colonies on each of them. With these data, we could compare the growth characteristics of mixed and pure colonies. We have shown that curve similarity metrics can be used to give a rough estimate of dominance of a given strain. This was validated using our DI for relative fluorescent area and growth curve similarity measurements.

We found that the social behavior of distant yeast strains is surprisingly complex, proving that even at the sub species level the different strains are quite divergent. These outcomes ranged from competitive exclusion to the formation of distinct core-ring structures, revealing a complex niche partitioning within the colony’s three-dimensional architecture (Figure 7B). These microscopy images suggest that it is not enough to analyze the 2D behavior of colonies, but it is important to study mixed colony growth in 3D. We also found that competitive outcomes are highly dependent on the environment, as the entire competitive hierarchy changed significantly when shifting from rich to minimal media (Figure 7), again underscoring a high degree of strain specialization. The method presented is useful to study interactions between various strains and just by quantifying the growth rates of colonies, the method can give a hint if one strain dominates the other one. The observed complex interactions and spatial structures appearing in mixed colonies also open new research lines as these underscore the need to study the observed colony shapes in three dimensions combining experiments and modeling.^61^

### Limitations of the study

Both growth curve-based and fluorescence-based methods have inherent experimental limitations. For growth curve analysis, a key limitation arises when the difference between competition outcomes is small relative to the noise of the growth curves. The acceptable signal-to-noise threshold will depend on the variability of the growth curves and the number of parallel colonies monitored per experiment. Additionally, in this study we excluded edge colonies (first and last rows and columns of the array). With rigorous validation, inclusion of edge colonies could potentially recover additional usable data.

Fluorescence-based methods present distinct limitations. First, labeling strains with fluorescent markers can affect cellular physiology. This means that labeled variants must be independently validated before conclusions can be extended to wild-type behavior. Second, overhead epifluorescence imaging of colonies is inherently limited by the depth of light penetration and objective depth of field. This optical limitation restricts spatial information to the colony surface and thus limits assessment of internal 3D colony architecture.

Our analysis here could capture mainly strain competition caused dominance patterns and could not capture in depth more complex social interactions between strains. Better characterization of the vertical structural distributions inside colonies and precise quantification of cell numbers and locations of each strain in a colony will enable more detailed characterization of social interactions among yeast strains.

## Resource availability

### Lead contact

Further information and requests for resources and reagents should be directed to and will be fulfilled by the lead contact, Csaba István Pongor (pongor.csaba.istvan@itk.ppke.hu).

### Materials availability

This study did not generate new unique reagents.

### Data and code availability

• All data reported in this paper will be shared by the lead contact upon request.
• All original code has been deposited at GitHub and is publicly available at https://github.com/CsikaszNagyLab/ColonyInsight/ as of the date of publication.
• Any additional information required to reanalyze the data reported in this paper is available from the lead contact upon request.

## Acknowledgments

We acknowledge support by the Hungarian 10.13039/501100018818National Research, Development and Innovation Office (10.13039/501100011019NKFI/NRDI) through the 10.13039/501100003549Hungarian Scientific Research Fund (OTKA-K20-134489 and ADVANCED24-149463) and University Research Scholarship Program (EKÖP-25-3-II-PPKE-57 and EKÖP-25-4-I-PPKE-22) of the Ministry for Culture and Innovation from the source of the National Research, Development and Innovation Fund.

## Author contributions

These authors contributed equally: T.G. and B.T.G.

Designing the study, T.G., B.T.G., B.P., V.M., C.I.P., and A.C.-N.; performing experiments and collecting data, T.G, B.P., C.I.P., and V.M. ; developing technical and software tools, B.T.G. and C.I.P.; preforming image analysis, B.T.G. and C.I.P.; analyzing data, T.G. and C.I.P.; writing - the first draft, T.G. and C.I.P; writing – review and editing, A.C.-N. and C.I.P.; supervision, A.C.-N. and C.I.P.

All authors contributed to revising the manuscript and approved the final version.

## Declaration of interests

The authors declare no competing interests.

## STAR★Methods

### Key resources table

**Table:** 

REAGENT or RESOURCE	SOURCE	IDENTIFIER
**Chemicals, peptides, and recombinant proteins**		

Yeast extract	Duchefa	Y1333, CAS number 8013-01-2
Peptone	Duchefa	P1328, CAS number 73049-73-7
Glucose	Molar Chemicals	02141, CAS number 5996-10-1
Agar	Duchefa	M1002, CAS number 9002-18-0
G-418	Duchefa	G0175, CAS number 108321-42-2
Yeast nitrogen base	BD Difco	233520
Monosodium glutamate	Sigma-Aldrich	49621, CAS number 6106-04-3
Yeast drop out mix	Sigma-Aldrich	Y1771

**Experimental models: Organisms/strains**		

*S. cerevisiae* Y55	N/A	ATCC: 52530
*S. cerevisiae* Sgu421	N/A	Landry et al.^15^
*S. cerevisiae* BY4743	N/A	ATCC: 4040005
*S. cerevisiae* YPS606	N/A	Liti et al.^13^

**Software and algorithms**		

Gphoto	^–^	http://www.gphoto.org
OpenCV	Bradski, G.^35^	https://opencv.org
Dtw-python	Giorgino^40^	https://pypi.org/project/dtw-python/
SimpleITK	Lowekamp, B. et al.^43^	https://simpleitk.org
Liquid Handling protocols,	this work	https://github.com/CsikaszNagyLab/Automated_Growth_Analysis
Image Processing Workflows	this work	
Image Segmentation workflow	this work	
Automated Image Acquisition Protocol (NIS Jobs)	this work	

**Other**		

Nunc™ OmniTray™ Single-Well Plate	Nunc	N/A

### Experimental model and study participant details

#### Yeast strains

All experiments were carried out using *S. cerevisiae* strains. For our studies we used two standard laboratory strains - Y55 ((13) and BY4743 (14) - and two wild strains - Sgu421 (15) and YPS606 (13). These strains were transformed using a KanMX cassette at the HO locus and labeled used constitutively expressed (Tef1 promoter) fluorescent markers (YFP and RFP). Overall, two variants were created for each strain HOyoYFP-KANMX and HOyoRFP-KANMX of the a/α haplotype.

### Method details

#### Culture conditions

All strains were cultured in standard YPD medium. In short: 1% yeast extract (Duchefa), 2% peptone (Duchefa)) dissolved in double-distilled water was autoclaved, and glucose (Molar Chemicals) was added afterward in 2% final concentration. Precultures were cultivated overnight with shaking and diluted to 0.5 OD600. Optical density was measured using a BioTek Synergy HTX plate reader.

During our experiments we used YPD and SD medium. For YPD plates, 2% agar (Duchefa) was added to YPD before autoclaving. Additionally, 100 μg/μL G-418 (Duchefa) was added to prevent contamination during lengthy experiments. For complete synthetic defined (SD) plates, we used double standard concentrations: 6.8 g/L yeast nitrogen base (Sigma-Aldrich), 4.4 g/L monosodium glutamate (Sigma-Aldrich), 200 mL 10x yeast drop-out mix (Sigma-Aldrich) and 2% agar (Duchefa). Plates were poured with defined volume of 50 mL for OmniTray (Nunc OmniTray Single-Well Plate cat.no: 242811). Media was measured with a serological pipette, and plates were placed on a leveled surface to ensure consistency. Plates were poured the day before the experiment and left to dry at room temperature for 24h. This later step is important as fresh plates do not absorb the culture droplets left when using the pinning tool (see later).

#### Liquid handling robot

Samples were prepared using a Hamilton Microlab Starlet Liquid handling system equipped with 4 1000 mL pipetting heads and Co-Re grippers to move the pinning tool. The system is also a laminar flow system as it is equipped with a HEPA filter. A custom-made pinning tool is used to transfer liquid cultures using 96 steel cylindrical ejector pins (from VP Scientific) with a diameter of 0.8 mm and 1.5 mm cylindrical head. The tool is made of two aluminum plates held together using M3 spacers. The top plate is used to grip the pinning tool, while the pins are mounted in an array of holes with a diameter of 1 mm. Protocols have been written in the Venus 4 (version 4.7.0.7744) development environment (see results section for details).

#### Imaging station

Time-lapse imaging was conducted in a custom 50-mm Styrofoam enclosure equipped with a Nikon D3200 DSLR and an 18-55 mm f/3.5–5.6 zoom lens. System automation was managed by a Raspberry Pi 5 (Raspberry Pi OS, Debian 12) using custom Python 3 scripts (zenodo repository and supplementary materials) utilizing the gphoto2 interface to control image acquisition and transistor-switched LED illumination. Internal temperature was monitored continuously via a DS18B20 digital sensor.

#### Growth plate preparation and handling

From the overnight cultures, a template plate was prepared for pining. This was prepared in 96-well plate in liquid phase using our two-step protocol found in our GitHub repository (see resource availability section of this paper). First, the 8 strains were mixed in standard 1.5 mL centrifuge tubes and dispensed in their final layout. The layout of the strains is shown in Figure S11. This plate is used to stamp the 12 agar plates (6 YPD and 6 SD) with the exact same layout using our protocol shared in our GitHub repository (see resource availability section of this paper). The plates are left to absorb the droplet, then transferred to the imaging station.

#### Microscopy

To validate our findings, we used fluorescent microscopy to visualize the fluorescent strains used in this study. For our experiments, we used a Nikon Ti2 E inverted microscope with a motorized filter turret and stage. To acquire the images, a Nikon CFI Plan Apochromat 2× objective with a numerical aperture of 0.1 was used with Nikon FITC filter cube for GFP and Nikon TRIC filter cube for RFP. For image acquisition, we used NIS Elements AR version 5.3 with the JOBS module for creating an automated measurement protocol (see GitHub repository and resource availability section of this paper).

### Quantification and statistical analysis

#### Growth curve analysis

Growth curves were extracted from time-lapse images of plates containing the grid pattern of colonies. These time-lapse images are processed in a two-stage Python pipeline: plate and colony detection. The plate detection workflow using OpenCV (36) was as follows: Images were converted to grayscale and uneven illumination were corrected by subtracting the morphological opening of the images (using a large 30-pixel structuring element) from the original image. Following thresholding, a distance transform is applied and ray-tracing is applied to locate rectangular plates. Extracted plate images were used in the colony identification step. Grid localization was achieved via a Fourier transform of row/column intensity sums. Regions of interest (ROI) were created per grid position using peak detection and Difference of Gaussians (DoG) for colony border delineation. Statistical descriptors (area and circularity) are extracted per colony per time frame.

For each sample (strain or co-culture), a per-plate mean growth curve was derived by averaging colony area values across central colonies at each time point (typically 3 colonies per sample per plate, with 6 replicate plates). Raw area data was filtered to remove outliers: a circularity threshold filter removed time-points where colony circularity fell below 0.85. Then a local rolling-window filter flagged individual time-series artifacts deviating more than 15% from a 10-point centered rolling average. After outlier removal, per-sample mean growth curves were derived from mean data by using a 10-point centered moving average. Edge colonies, defined as the outermost rows and columns of a plate, were excluded from further analysis.

#### Curve similarity analysis

To quantify the similarity between co-culture and mono-culture growth dynamics mean growth curves were used (see above). Both compared curves were reindexed to a common time axis followed by Dynamic Time Warping (DTW) to determine curve distance *d*. The DTW distance was converted to a similarity score using the inverse transform *s* = 1/(1 + *d*). For each co-culture, DTW similarity was calculated against both constituent mono-culture curves across all replicate plates. DTW similarity scores were calculated per plate, yielding *n* = 6 data points per condition (one per replicate plate). Individual data points in violin plots represent per-plate similarity scores. Similarity metrics of the two strains in each pairing were compared using Welch’s *t* test; ns = not significant, ∗*p* < 0.05, ∗∗*p* < 0.01, ∗∗∗*p* < 0.001.

#### Microscopy image analysis

Images were processed in two steps. First, a global colony mask was generated by removing low-intensity background from each fluorescence channel using a percentile-based threshold, combining and binarizing the cleaned channels. Each channel was then individually corrected for uneven illumination by dividing by a Gaussian-blurred background estimate (σ = 100 pixels) and segmented using the Moments auto-thresholding method as implemented in SimpleITK (35). Using the segmented channels we have determined the relative area of the green and red strains compared to the total colony area in the top layers of the colonies. Relative fluorescent area was quantified per colony (*n* = 4 colonies per strain pairing) from one plate per condition. Data are represented as violin plots. Groups were compared using Welch’s *t* test; ns = not significant, ∗*p* < 0.05, ∗∗*p* < 0.01, ∗∗∗*p* < 0.001.

### Additional resources

All original code has been deposited at: https://github.com/CsikaszNagyLab/ColonyInsight/ and will be publicly available as of the date of publication. Our repository contains all necessary scripts and instructions for reproducing our image processing methods. The following has been deposited: sample preparation protocols (using Hamilton Venus), image acquisition scripts, image processing scripts (with 4 example images), and microscopy image acquisition workflow (using Nikon Jobs). The repository also contains information on installation and use of software components.
