# Supplementary material for: ColonyInsight automated workflow reveals complex dominance in mixed yeast colonies
Source: iScience. 2026 Jul 22;29(8):116639. doi: 10.1016/j.isci.2026.116639 (PMC13427406; doi:10.1016/j.isci.2026.116639)
Supplement: Document S1. Figures S1–S12 [file mmc1.pdf]

iScience, Volume *29*

Supplemental information

ColonyInsight automated workflow reveals complex dominance in mixed yeast colonies

Tünde Gaizer, Bence T. Gaizer, Bíborka Pillér, Valentina Madár, János Juhász, Attila Csikász-Nagy, and Csaba I. Pongor

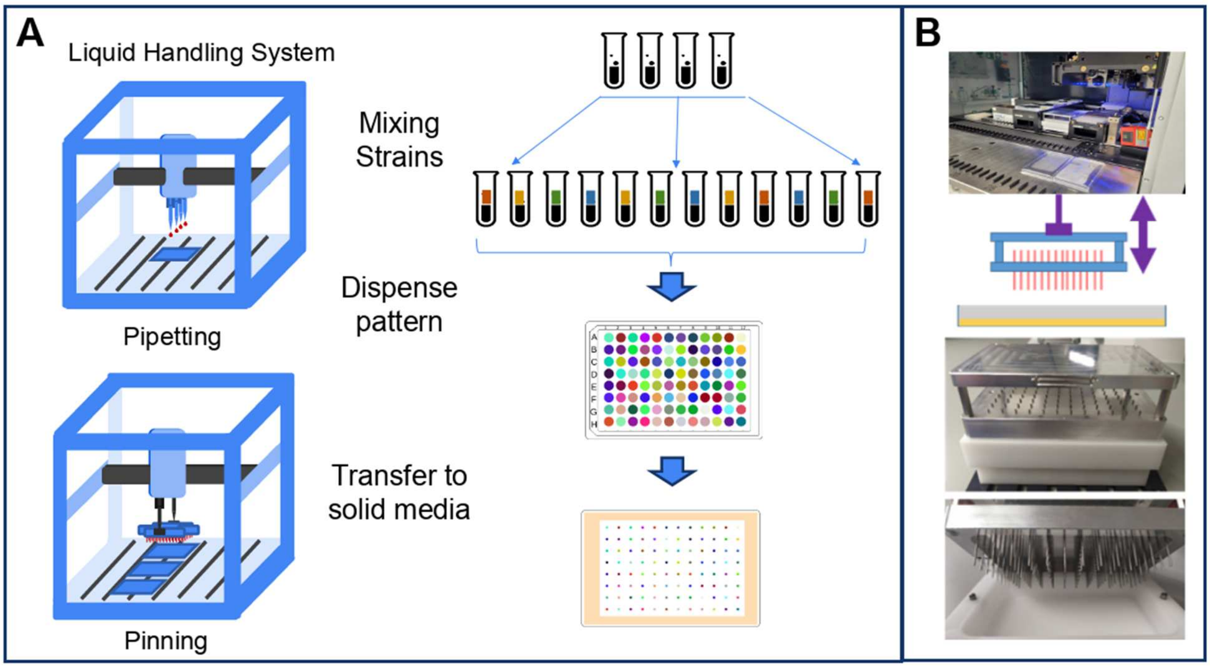

Figure S1. Overview of the sample preparation protocol. (A) Graphical summary of the sample preparation protocol. (B) Overview of the pinning procedure and the custom pin tool.

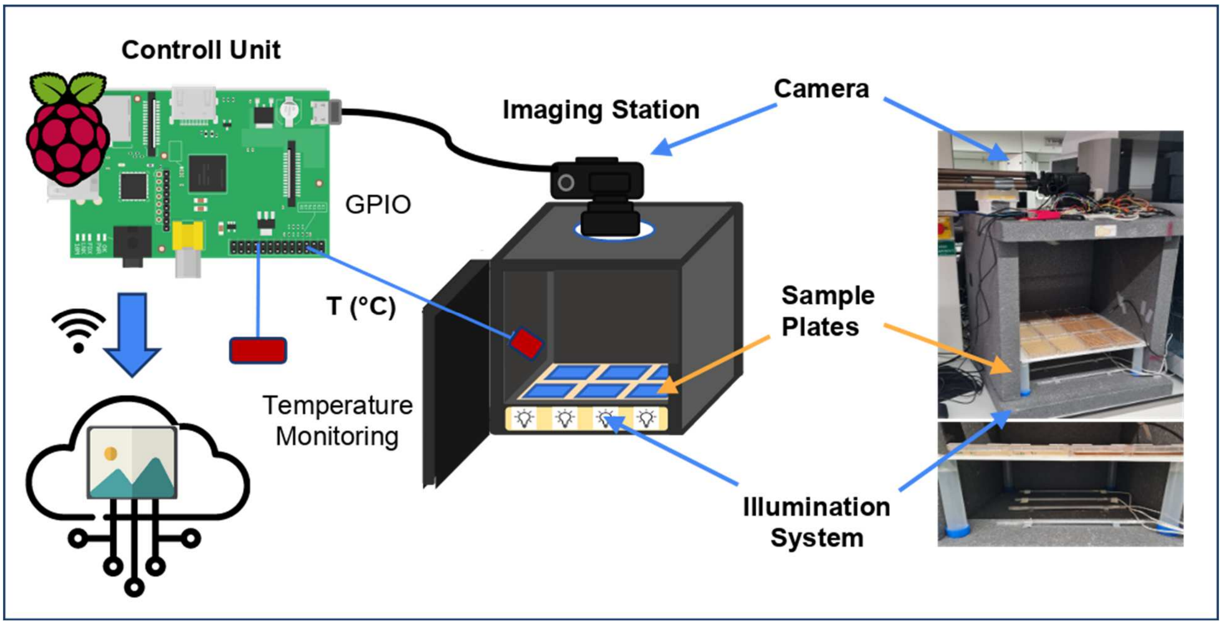

Figure S2. Overview of the Imaging Station.

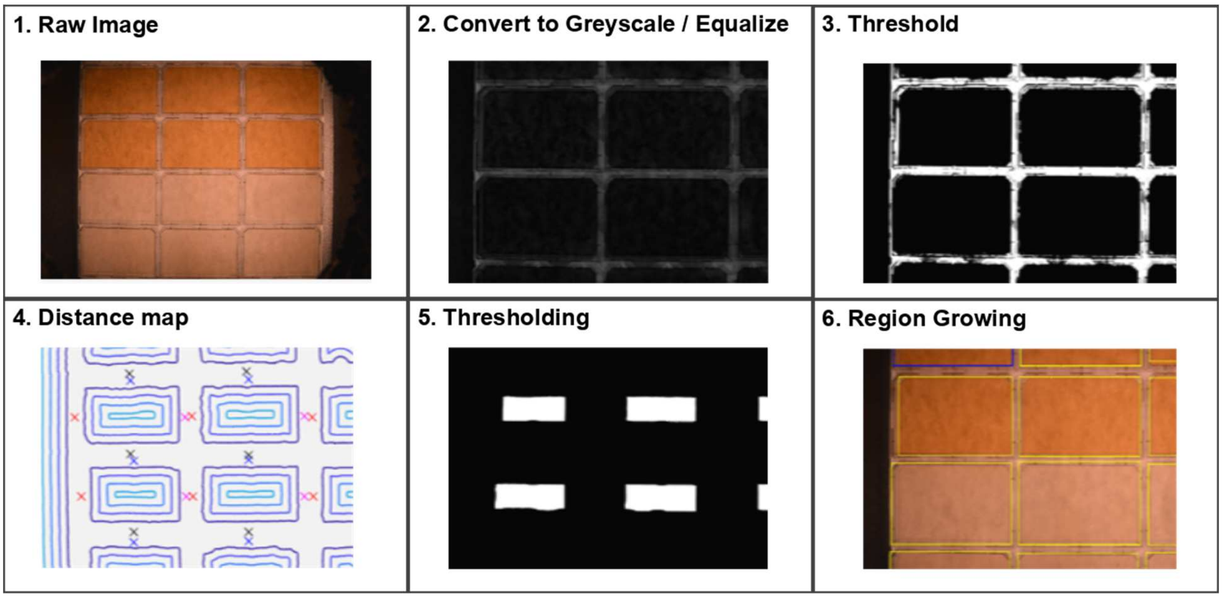

Figure S3. Overview of the image processing workflow to identify individual plates. First, it transforms the image to grayscale and uses light equalization to remove artifacts from uneven lighting. Next, it applies a threshold to find segments of the plate borders, then employs a distance transform followed by another thresholding to select central area of each plate (seeds). From the seeds identified in the previous step, the border of the plates is identified using a ray-tracing algorithm.

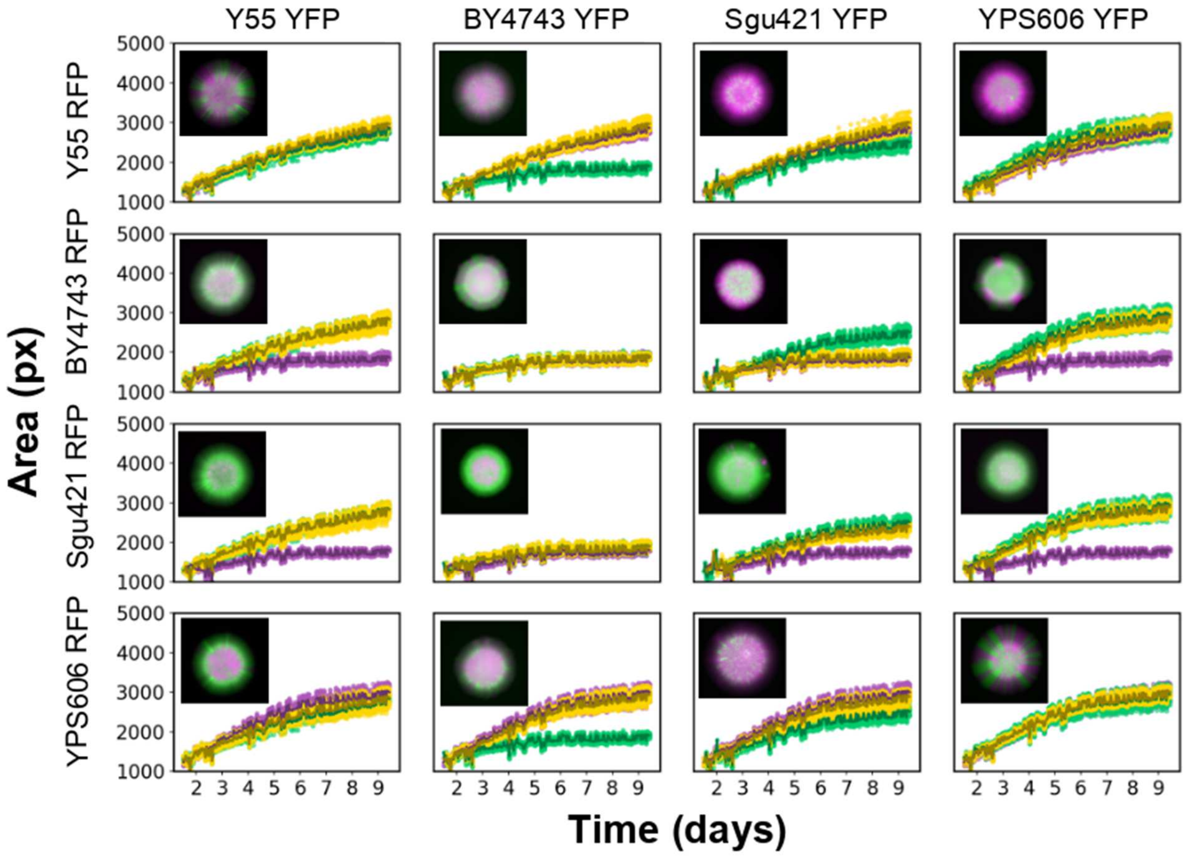

Figure S4. Overview of growth-curve data and microscopy images on SD. A comprehensive overview of competitive interactions between the four differently labeled yeast strains (Y55, BY4743, Sgu421, YPS606), each labeled with either Yellow Fluorescent Protein (YFP) or Red Fluorescent Protein (RFP), grown on YPD. Each subplot corresponds to a specific pairing and within each subplot, scatter points show individual colony growth curves pooled across all replicate plates (n = 6 plates per condition; approximately 2–3 central colonies per plate per sample). The solid line represents the mean growth curve computed across all non-outlier central colonies from all plates combined, smoothed with a 10-point centered rolling average. Each subplot also shows a selected two channel microscopy image after 9 days of growth where magenta corresponds to RFP and the green channel corresponds to the YFP signal.

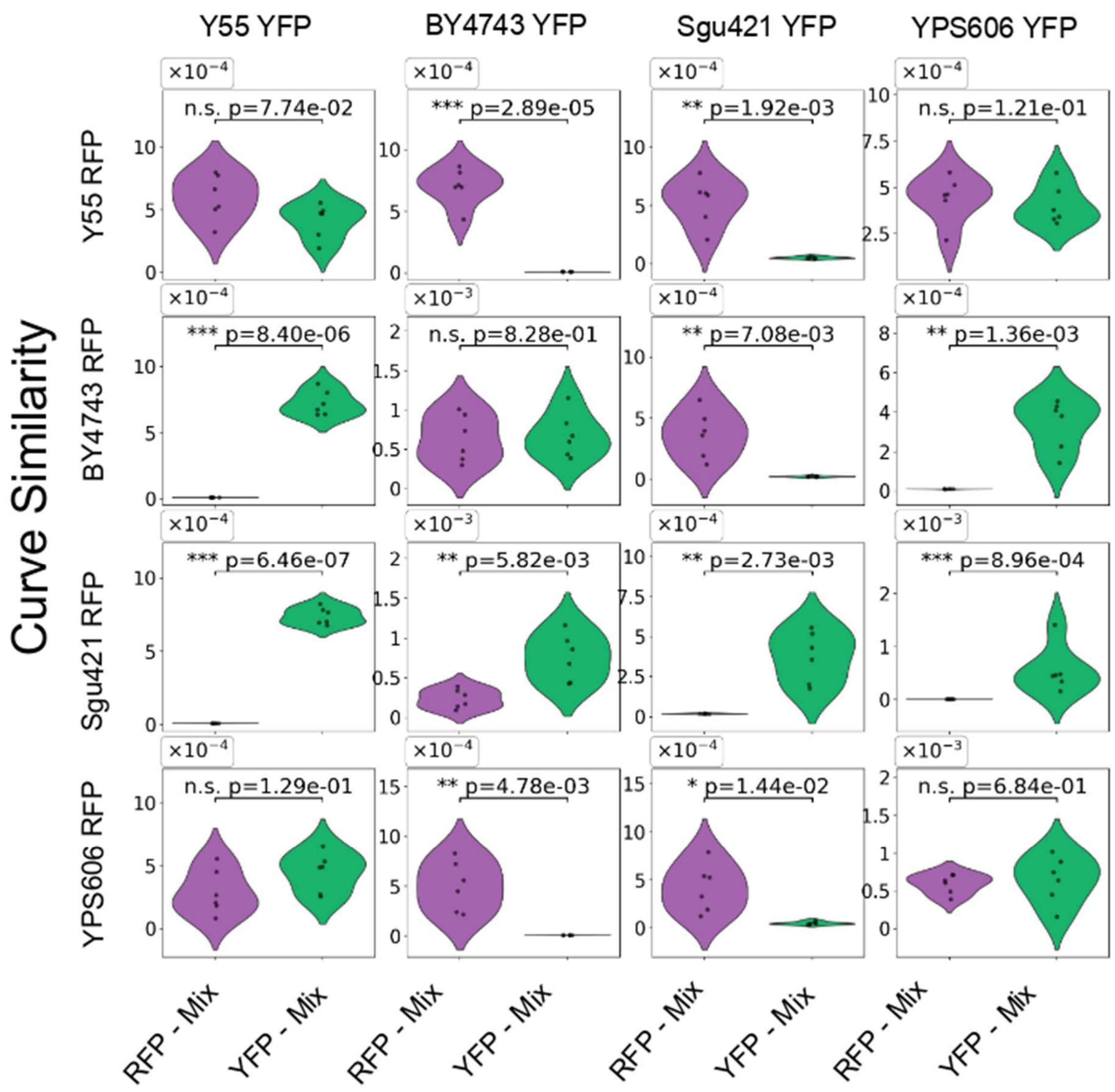

Figure S5. Overview of dynamic time warping (DTW) curve similarity metric (*s*) on SD. A comprehensive grid-plot visualizing DTW based similarity metric. Each subplot corresponds to a specific pairing, with the row indicating the RFP-labeled strain and the column indicating the YFP-labeled strain. On each subplot the mixed colony's growth curve is compared to the growth curve of both of its constituent strains. Each data point represents the DTW-based similarity score calculated between the per-plate mean growth curve of the co-culture and that of the respective monoculture (n = 6 replicate plates per condition). Per-plate mean curves were derived by averaging colony area across central colonies at each time point, followed by 10-point centered rolling average smoothing. Violin shapes show the distribution of per-plate similarity scores. Groups were compared using Welch's t-test; ns = not significant, *p < 0.05, **p < 0.01, ***p < 0.001.

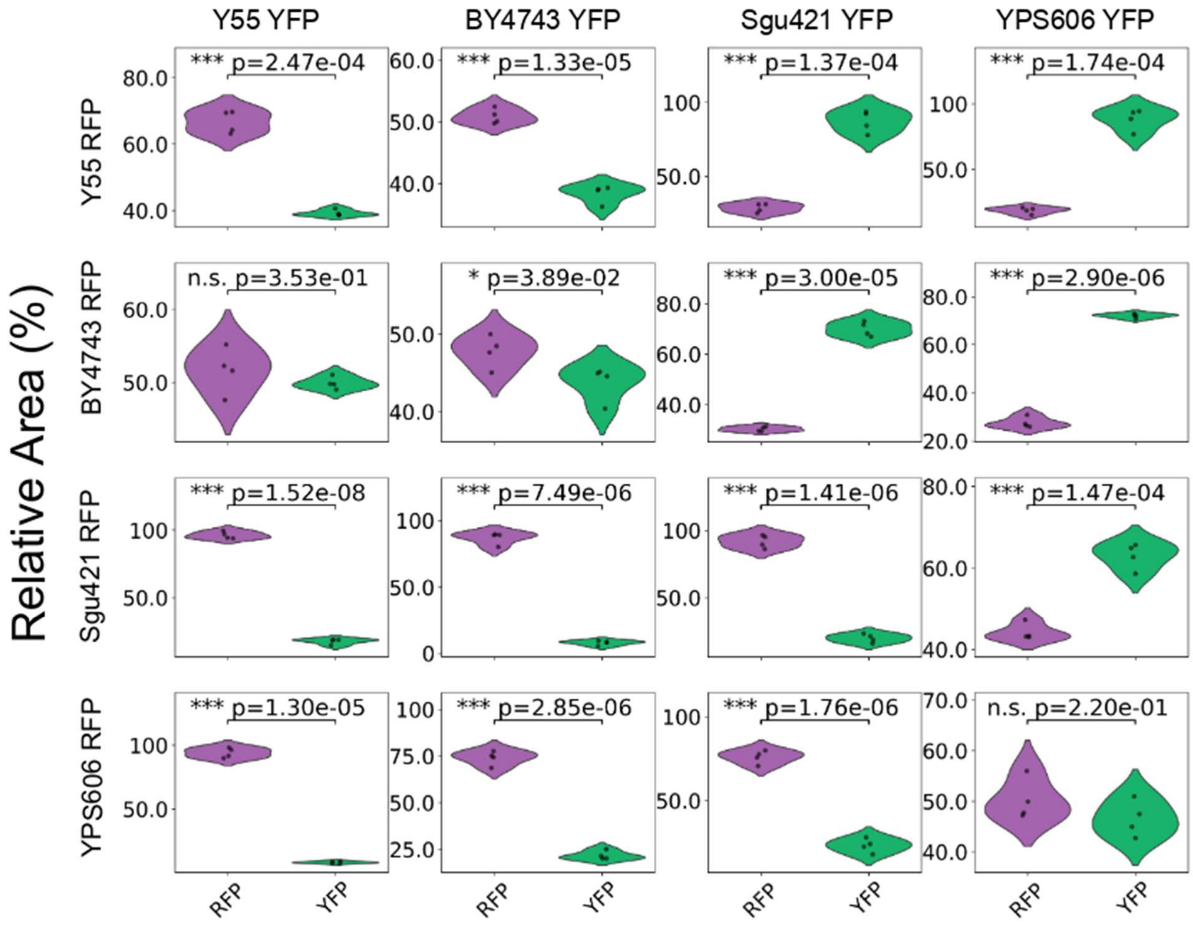

Figure S6. Analysis of Fluorescent Microscopy Data on YPD. Summary of the Relative Area of Components in mixed colonies using Fluorescent Microscopy on YPD. Each mixed colony contains two of the four different labeled yeast strains (Y55, BY4743, Sgu421, YPS606) labeled with Yellow Fluorescent Protein (YFP) or Red Fluorescent Protein (RFP). Each subplot corresponds to a specific pairing, with the row indicating the Red Fluorescent Protein (RFP) labeled strain and the column indicating the Yellow Fluorescent Protein (YFP) labeled strain. Relative area is the area of the segmented label with respect to the area of the whole colony. Violin plots appertaining to RFP strain is shown in magenta, while YFP is shown in green for each pairing (each violin element is derived from 4 individual colonies). Relative fluorescent area was quantified per colony (n =4 colonies per strain pairing) from one plate per condition. Data are represented as violin plots. Groups were compared using Welch's t-test; ns = not significant, *p < 0.05, **p < 0.01, ***p < 0.001.

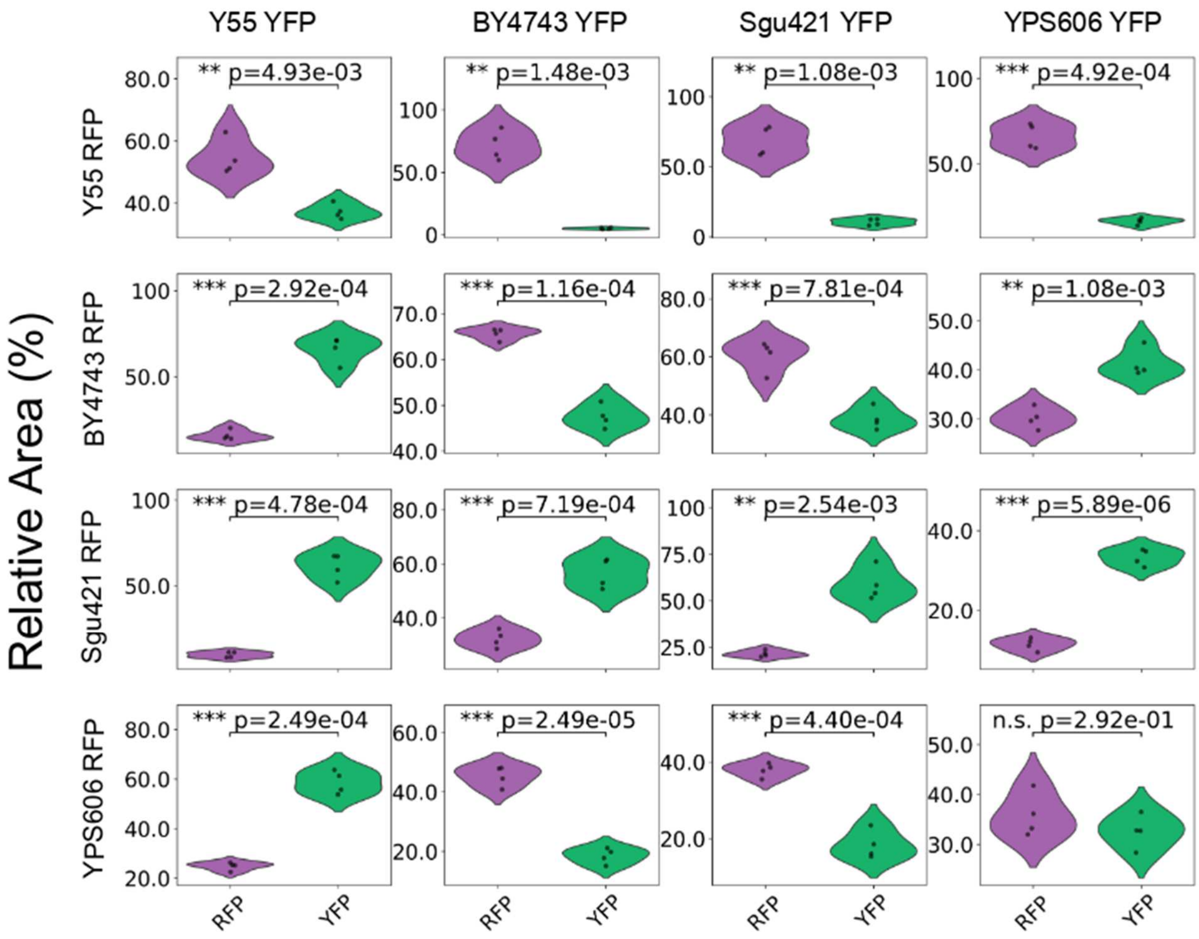

Figure S7. Analysis of Fluorescent Microscopy Data on SD. Summary of the Relative Area of Components in mixed colonies using Fluorescent Microscopy on YPD. Each mixed colony contains two of the four different labeled yeast strains (Y55, BY4743, Sgu421, YPS606) labeled with Yellow Fluorescent Protein (YFP) or Red Fluorescent Protein (RFP). Each subplot corresponds to a specific pairing, with the row indicating the Red Fluorescent Protein (RFP) labeled strain and the column indicating the Yellow Fluorescent Protein (YFP) labeled strain. Relative area is the area of the segmented label with respect to the area of the whole colony. Violin plots appertaining to RFP strain is shown in magenta, while YFP is shown in green for each pairing (each violin element is derived from 4 individual colonies). Relative fluorescent area was quantified per colony (n =4 colonies per strain pairing) from one plate per condition. Data are represented as violin plots. Groups were compared using Welch's t-test; ns = not significant, *p < 0.05, **p < 0.01, ***p < 0.001.

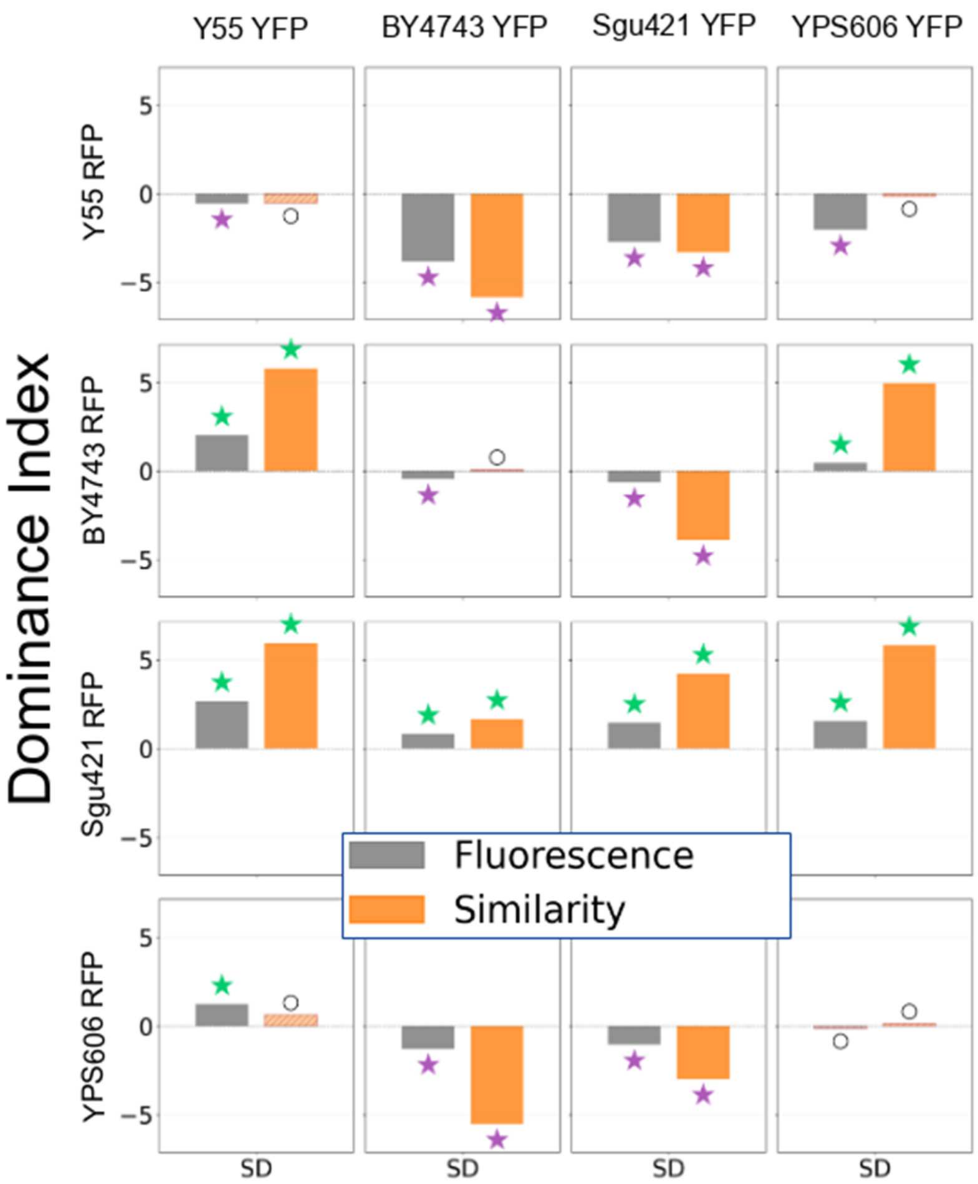

Figure S8. Grid Plot for Dominance Indices on YPD). Each subplot within this grid directly compares the dominance indices for a single strain pair. The Dominance Index (DI) is calculated as the log2 ratio of either the growth curve similarity scores or the relative fluorescent areas of the competing YFP and RFP strains within a co-culture. This visualization classifies each result based on the sign of the dominance index and interactions are deemed as neutral if there is no significant difference with p-value>0.05 (Welch's t-test). A significant or above-threshold dominant strain is designated by a star symbol (★), while a neutral interaction is marked with an open circle (○). Negative values correspond to dominance by the RFP, positive values by the YFP expressing strain, represented by the colors of the significance stars.

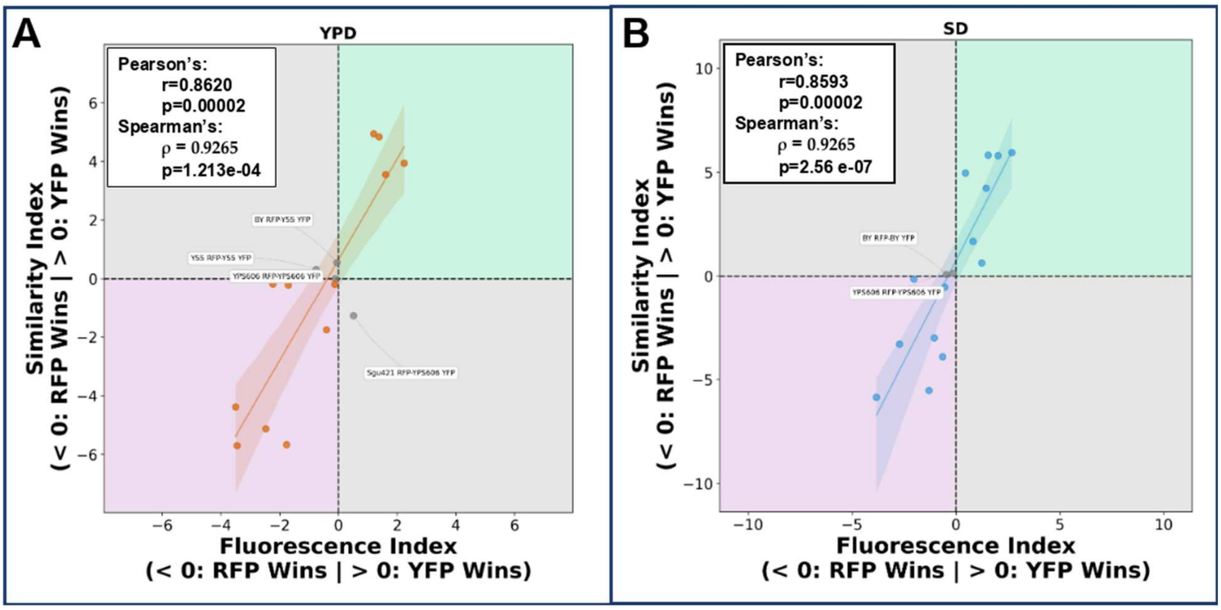

Figure S9. Figure shows the correlation scatter plots between the dominance indices calculated for all the 16 investigated strain pairs (panel A for YPD and Panel B for SD). The strength and significance of the linear relationship are quantified using Pearson's correlation coefficient (r), Spearman's ranked correlation, with 95% confidence intervals derived from Fisher's z-transformation. The plot area is segmented into four quadrants to visualize agreement of the methods. The top-right (both methods show the dominance of the YFP strain) and bottom-left (both show dominance of the RFP strain) quadrants are shaded to indicate concordance. The off-diagonal quadrants are shaded gray to highlight discordant results where the two methods disagree.

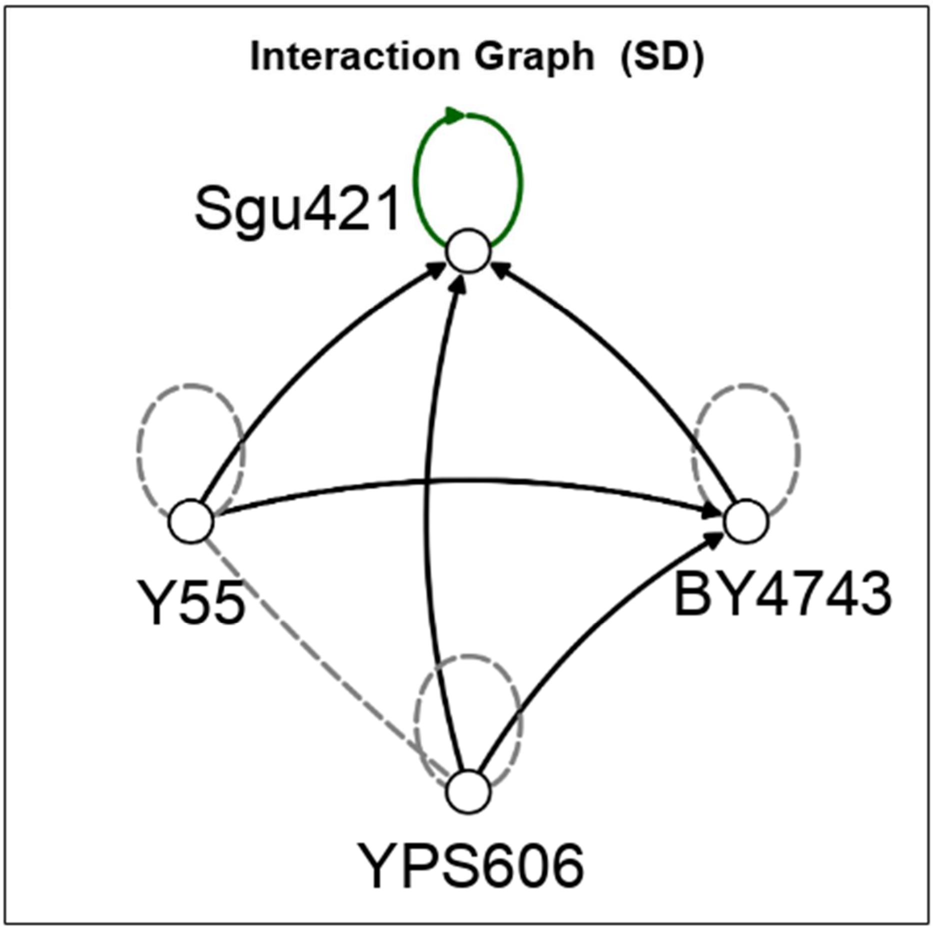

Figure S10. Summary of dominance hierarchies and spatial patterns on SD. (A) Interaction graph. Nodes represent interacting partners. The edges connecting the nodes visually encode the outcome of the competition. Directed, arrows are drawn from the dominant strain to the less dominant partner. Neutral interactions are rendered as a gray dashed line, for cases where dominance indexes are indistinguishable using $p>0.05$, Welch's t-test). Black arrows indicate a consensus outcome where both reciprocal labeling combinations (RFP vs. YFP and YFP vs. RFP) agree on the direction of dominance. Colored arrows (red or green) indicate cases where the reciprocal labeling combinations differ; in these instances of disagreement, the edge color reflects the specific color of the dominant strain in that combination. (**B)** Representative microscopy images of mixed colonies. Images were taken at day 4, day 7 and day 9.

	1	2	3	4	5	6	7	8	9	10	11	12
A	Y55 RFP	Y55 YFP	BY RFP	BY YFP	Sgu421 RFP	Sgu421 YFP	YPS606 RFP	YPS606 YFP	Y55 RFP-Y55 YFP	BY RFP-BY YFP	Sgu421 RFP-Sgu421 YFP	YPS606 RFP-YPS606 YFP
B	Sgu421 YFP	Y55 RFP-BY YFP	BY RFP-Sgu421 YFP	Y55 RFP	YPS606 RFP	BY RFP-Sgu421 YFP	Y55 RFP-BY YFP	BY YFP	BY RFP-BY YFP	YPS606 RFP-BY YFP	BY RFP-Y55 YFP	YPS606 RFP
C	Sgu421 RFP	Y55 RFP-Sgu421 YFP	BY RFP-YPS606 YFP	Y55 YFP	YPS606 YFP	BY RFP-YPS606 YFP	Y55 RFP-Sgu421 YFP	Sgu421 RFP	Sgu421 RFP-Sgu421 YFP	Sgu421 RFP-YPS606 YFP	Sgu421 RFP-Y55 YFP	YPS606 YFP
D	BY YFP	Y55 RFP-YPS606 YFP	Sgu421 RFP-BY YFP	BY RFP	Y55 RFP-Y55 YFP	Sgu421 RFP-BY YFP	Y55 RFP-YPS606 YFP	Sgu421 YFP	YPS606 RFP-YPS606 YFP	YPS606 RFP-Sgu421 YFP	YPS606 RFP-Y55 YFP	Y55 RFP-Y55 YFP
E	BY RFP	BY RFP-Y55 YFP	YPS606 RFP-BY YFP	BY YFP	BY RFP-BY YFP	YPS606 RFP-BY YFP	BY RFP-Y55 YFP	Y55 RFP	YPS606 RFP	BY RFP-Sgu421 YFP	Y55 RFP-BY YFP	BY RFP-BY YFP
F	Y55 YFP	Sgu421 RFP-Y55 YFP	Sgu421 RFP-YPS606 YFP	Sgu421 RFP	Sgu421 RFP-Sgu421 YFP	Sgu421 RFP-YPS606 YFP	Sgu421 RFP-Y55 YFP	Y55 YFP	YPS606 YFP	BY RFP-YPS606 YFP	Y55 RFP-Sgu421 YFP	Sgu421 RFP-Sgu421 YFP
G	Y55 RFP	YPS606 RFP-Y55 YFP	YPS606 RFP-Sgu421 YFP	Sgu421 YFP	YPS606 RFP-YPS606 YFP	YPS606 RFP-Sgu421 YFP	YPS606 RFP-Y55 YFP	BY RFP	Y55 RFP-Y55 YFP	Sgu421 RFP-BY YFP	Y55 RFP-YPS606 YFP	YPS606 RFP-YPS606 YFP
H	Y55 RFP-BY YFP	Y55 RFP-Sgu421 YFP	Y55 RFP-YPS606 YFP	BY RFP-Y55 YFP	Sgu421 RFP-Y55 YFP	YPS606 RFP-Y55 YFP	BY RFP-Sgu421 YFP	BY RFP-YPS606 YFP	Sgu421 RFP-BY YFP	YPS606 RFP-BY YFP	Sgu421 RFP-YPS606 YFP	YPS606 RFP-Sgu421 YFP

Figure S11. Layout of the experiments. The samples located at each location are provided. Mixed samples are separated by a "-" dash. Sample allocation was randomized by placing each sample both in central and edge positions.

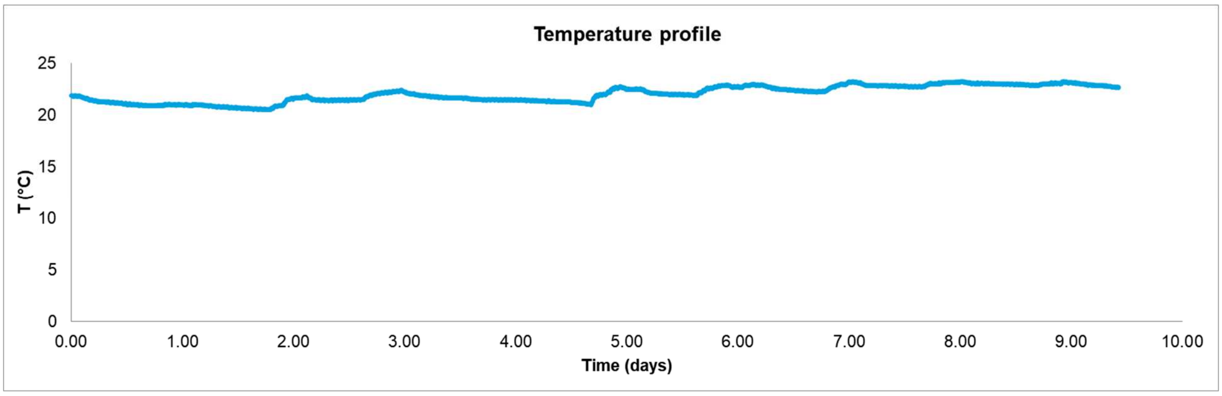

Figure S12. Temperature profile during the experiment. The temperature inside the imaging station was logged every 5 minutes during the experiment.
